# IL-12p40 drives oral squamous cell carcinoma progression via MMPs and IL-12Rβ1/STAT3 signaling

**DOI:** 10.1186/s12935-026-04413-0

**Published:** 2026-07-16

**Authors:** Dongxiao Tang, Shaoqin Tu, Yiting Shao, Yuxuan Wang, Nan Wei, Hongkun Sun, Yi Feng, Rui Zhang, Zheng Chen

**Affiliations:** 1 Department of Stomatology, The Third Affiliated Hospital, Sun Yat-sen University, Guangzhou, 510630 Guangdong China; 2 Department of Anesthesiology, State Key Laboratory of Ophthalmology, Zhongshan Ophthalmic Center, Sun Yat-sen University, Guangzhou, 510060 Guangdong China

**Keywords:** Oral squamous cell carcinoma, IL-12p40, IL-12Rβ1, STAT3 signaling, MMP2/MMP9

## Abstract

**Background:**

Oral squamous cell carcinoma (OSCC) is an aggressive head and neck malignancy with frequent lymphatic metastasis and poor clinical outcomes. IL-12p40, a shared subunit of IL-12 and IL-23, can engage IL-12Rβ1, but its role in OSCC tumor-cell responses remains unclear. This study investigated whether IL-12p40 promotes OSCC progression and explored the underlying IL-12Rβ1/STAT3/MMP pathway.

**Methods:**

IL-12p40 expression was examined by immunohistochemistry in 90 OSCC tissues and 60 adjacent oral mucosa samples, and serum IL-12p40 was measured in 30 patients and 15 healthy controls. SCC9 and CAL33 cells were treated with recombinant IL-12p40, anti-IL-12p40 antibody, Stattic, or GM6001. Proliferation, wound healing, migration, invasion, and colony formation were assessed. RNA sequencing, quantitative real-time PCR, western blotting, IL-12Rβ1 silencing, and shRNA-mediated STAT3 knockdown were used for mechanistic validation. Subcutaneous xenograft, orthotopic tongue tumor, and lymphatic metastasis models were established. Statistical analyses included t tests, analysis of variance, chi-square or Fisher’s exact tests, Kaplan-Meier analysis, Cox regression, and receiver operating characteristic analysis.

**Results:**

IL-12p40 was elevated in OSCC tissues and patient serum. High tissue IL-12p40 expression was associated with lymph-node metastasis and poorer overall survival. IL-12p40 enhanced OSCC cell proliferation, migration, invasion, and colony formation in vitro and promoted tumor growth and lymphatic dissemination in vivo. Mechanistically, IL-12p40 activated IL-12Rβ1/STAT3 signaling and increased MMP2/MMP9 expression. IL-12Rβ1 silencing, STAT3 inhibition, STAT3 knockdown, and GM6001-mediated MMP blockade attenuated these effects.

**Conclusions:**

IL-12p40 promotes OSCC progression through an IL-12Rβ1/STAT3/MMP-related pathway, suggesting a candidate mechanism that warrants further therapeutic validation.

**Supplementary Information:**

The online version contains supplementary material available at https://doi.org/10.1186/s12935-026-04413-0.

## Introduction

Oral squamous cell carcinoma (OSCC) remains a prevalent and lethal head-and-neck malignancy with stagnant survival in advanced disease despite multimodal therapy [1]. Although risk-factor control and earlier detection benefit subsets of patients [2], recurrence and regional lymph-node metastasis are common [3], underscoring the need to define tumor-intrinsic cues that couple inflammation to invasion and dissemination.

The OSCC tumor immune microenvironment (TIME) is frequently immunosuppressive and myeloid-rich, shaped by stromal-immune-epithelial crosstalk and cytokine networks that modulate both epithelial behavior and anti-tumor immunity [4]. Compared with other head-and-neck sites, such as the oropharynx and nasopharynx, oral cavity tumors often show distinct immune composition and stromal reprogramming that may blunt responses to PD-1/PD-L1 blockade and related strategies [5, 6]. While immune checkpoint inhibitors have transformed care in selected settings, overall response rates remain suboptimal, motivating efforts to map cytokine-driven remodeling of the OSCC TIME [7, 8].

The interleukin-12 (IL-12) family, including IL-12, IL-23, IL-27, IL-35, and IL-39, regulates innate and adaptive immunity and shapes anti-tumor responses [9, 10]. IL-12 promotes Th1 differentiation and cytotoxic programs, and IL-12/IL-23 signal through the shared receptor chain IL-12Rβ1 [11]. The shared p40 subunit (IL-12p40) exists as a monomer, homodimer, or heterodimer with p35 or p19, each with distinct functions [12]. Beyond classical immunology, dysregulated p40 is linked to autoimmunity, infection, and cancer, and tumor-cell-intrinsic p40 production has been reported [13]. In prostate cancer, elevated circulating p40 monomer correlates with progression, and p40 neutralization restrains tumor growth while enhancing IL-12/IFN-γ responses [14, 15]. Similar p40-dependent pro-tumor activity, with growth suppression upon selective p40 neutralization, has been shown in pancreatic ductal adenocarcinoma and triple-negative breast cancer [16, 17]. Together with contemporary insights that IL-12/IL-23 receptors engage JAK2/TYK2 to activate STATs [18, 19], these observations motivated us to test whether IL-12p40 signals via IL-12Rβ1 to activate STAT3 and MMP-linked invasion in OSCC.

Here, we investigate the role of IL-12p40/IL-12Rβ1 signaling in OSCC progression, with a focus on tumor-cell responses to IL-12p40 stimulation. Using patient tissues, we assessed clinicopathologic associations; in sera, we performed a case-control comparison between OSCC patients and healthy individuals. *In vitro*, we examined how recombinant IL-12p40 affected proliferation, migration, and invasion; and in vivo, we evaluated tumor growth and lymphatic spread. Unbiased transcriptomics and targeted perturbations, including IL-12Rβ1 silencing, STAT3 inhibition/knockdown, and MMP blockade, converged on an IL-12Rβ1-STAT3-associated program with up-regulation of MMP2/MMP9 in OSCC cells [20, 21]. Because IL-12p40 can exist as distinct molecular species, including monomer, homodimer, and IL-12/IL-23 heterodimers, clinical measurements of IL-12p40 in this study should be interpreted as total p40-related signals unless otherwise specified [11, 12].

Collectively, our data support a model in which IL-12p40 is associated with adverse OSCC progression and can reinforce pro-migratory and pro-invasive programs in OSCC cells through an IL-12Rβ1/STAT3/MMP-related axis. These findings provide mechanistic insight into cytokine-linked tumor progression and nominate this pathway for further therapeutic validation in OSCC.

## Materials and methods

### Cell culture and transfection

SCC9 and CAL33 OSCC cell lines were obtained from the American Type Culture Collection (ATCC), and HOK cells were purchased from ScienCell. Cell identity was verified by short tandem repeat profiling. SCC9 cells were cultured in DMEM/F12, and CAL33 cells in DMEM, both supplemented with 10% FBS and 1% penicillin/streptomycin at 37 °C in 5% CO_2_.

For cytokine stimulation, cells were treated with recombinant human IL-12p40/IL12B protein (Sino Biological, 10052-H08H; 20 ng/mL) unless otherwise indicated. For IL-12p40 neutralization, cells were treated with IL-12p40 together with an anti-IL-12p40 neutralizing antibody at the indicated concentration. For STAT3 inhibition, cells were pretreated with Stattic (10 µM) [22–24] for 1 h and maintained in Stattic-containing medium during the experiment. For MMP inhibition, cells were treated with GM6001 (10 µM) alone or in combination with IL-12p40 during Matrigel-coated Transwell invasion assays. Detailed information on recombinant proteins, inhibitors, antibodies, and working concentrations is provided in Table S8.

IL-12Rβ1 siRNAs were purchased from RiboBio (Guangzhou, China). Three siRNAs were screened, and the most effective siRNA was used for subsequent experiments. For STAT3 knockdown, three STAT3-targeting shRNA constructs and a non-targeting control shRNA were obtained from GeneChem (Shanghai, China). Lentiviral transduction was performed according to the manufacturer’s protocol, followed by puromycin selection. Knockdown efficiency was verified by qRT-PCR and Western blotting, and the most effective shSTAT3 construct was used for downstream assays. The siRNA and shRNA sequences are listed in Table S6.

### Patients and specimens

A total of 90 OSCC tumor specimens and 60 adjacent noncancerous tissues (2 cm from the tumor margin) were collected from patients diagnosed at the Department of Oral and Maxillofacial Surgery, Third Affiliated Hospital of Sun Yat-sen University between 2020 and 2022. Overall survival (OS) time was calculated from diagnosis to January 2025 (or death). Patients who had received preoperative chemotherapy or radiotherapy were excluded. Serum samples were obtained from an independent cohort consisting of 30 newly diagnosed, treatment-naive OSCC patients and 15 healthy individuals, and IL-12p40 concentrations were measured by ELISA.

All human samples were obtained with informed consent and processed according to the Declaration of Helsinki. The study was approved by the Institutional Review Board of the Third Affiliated Hospital of Sun Yat-sen University ([2020]02-017-01).

### Quantitative real-time PCR (qRT‒PCR)

Total RNA was isolated using TRIzol (Invitrogen, Carlsbad, CA). cDNA synthesis was performed with PrimeScript RT Master Mix (Takara, Kyoto, Japan). qPCR was run on a StepOnePlus Real-Time PCR System (Thermo Fisher, Waltham, MA) using SYBR Green master mix. Gene expression was normalized to 36B4 or GAPDH (as specified for each target) and calculated by the ΔΔCt method. Primer sequences are listed in Table S7.

### Western blotting

Cells were lysed in ice-cold RIPA buffer (Sigma-Aldrich, St. Louis, MO). Equal amounts of protein (30 µg) were separated by SDS-PAGE and transferred to PVDF membranes. After blocking with 3% BSA, membranes were incubated with primary antibodies overnight at 4 ℃, followed by HRP-conjugated secondary antibodies and ECL detection. Primary antibodies included IL-12p40 (Abcam, ab106270), IL-12Rβ1 (Affinity, DF2508), MMP9 (Affinity, AF5228), MMP2 (Affinity, AF5330), STAT3 (Bioworld, AP0366), p-STAT3 (Bioworld, BS4180), and GAPDH (Cell Signaling Technology, #2118). For STAT3 knockdown validation, STAT3, p-STAT3, MMP2, and MMP9 were examined in shNC- and shSTAT3-transduced cells treated with or without IL-12p40. Detailed antibody information and working dilutions are provided in Table S8.

### RNA-seq

SCC9 cells were revived, passaged, and seeded into six-well plates, then assigned to control (0 ng/mL IL-12p40) or treatment (20 ng/mL IL-12p40) groups. After 48 h stimulation, cells were lysed in 1 mL TRIzol and stored at -80 ℃. Total RNA integrity and concentration were assessed using the Agilent 2100 with the RNA Nano 6000 kit. Poly(A) mRNA was enriched with oligo(dT) magnetic beads, fragmented, and used for first-strand cDNA synthesis with random primers; second-strand cDNA was synthesized, end-repaired, A-tailed, adapter-ligated, size-selected, and PCR-amplified to generate libraries. Library quality was evaluated by Qubit 3.0 (quantification), Agilent 2100 Bioanalyzer (insert size), and Bio-Rad CFX96 qPCR (effective concentration > 10 nM). Qualified libraries were sequenced on an Illumina platform.

Raw reads were filtered to remove adapters and low-quality bases to obtain clean reads. Gene expression was estimated as FPKM. Differential expression was analyzed with DESeq2, using |log_2_ fold change| ≥ 1 and q < 0.05 to define DEGs. Functional enrichment of DEGs employed clusterProfiler for GO (MF/CC/BP; with gene-length bias correction) and KEGG pathway analyses.

### Immunohistochemistry (IHC) staining

FFPE human and mouse tumor specimens were sectioned, deparaffinized, rehydrated, and subjected to antigen retrieval. After endogenous peroxidase quenching, slides were blocked with 5% BSA for 30 min and incubated overnight at 4 ℃ with primary antibodies against IL-12p40, p-STAT3, MMP9, or MMP2, as appropriate. Secondary antibody incubation, DAB development, and hematoxylin counterstaining were performed according to standard protocols. IL-12p40 IHC in clinical samples was scored using an H-score system (0–300), calculated as the sum of staining intensity (0–3) multiplied by the percentage of positive cells [25, 26]. For mouse tumor tissues, p-STAT3, MMP9, and MMP2 staining was used to assess pathway activation. Detailed antibody information and working dilutions are provided in Table S8.

### Enzyme linked immunosorbent assay (ELISA)

Serum was collected from 30 OSCC patients and 15 healthy individuals at the Third Affiliated Hospital of Sun Yat-sen University (IRB [2020]02-017-01). After preparing sample/wash buffers and standards, ELISA was performed according to the manufacturer’s instructions: standards and samples were added to coated wells, incubated, washed, and sequentially incubated with biotinylated antibody, enzyme conjugate, substrate, and stop solution. OD_450_ was recorded to quantify IL-12p40 concentrations.

### Cell-based functional assays

Cell proliferation (CCK-8). Cells were seeded at 3 × 10^3^ cells/well in 96-well plates and cultured for the indicated times. CCK-8 reagent (Dojindo) was added for 2 h, and absorbance was read at 450 nm on a BioTek microplate reader.

Wound-healing assay. Cells were plated in six-well plates and serum-starved (FBS-free) to confluence. A linear scratch was made with a sterile 200-µL tip, then cultures were switched to 2% FBS medium. Images were captured at 0 h and 24 h in three random fields using a Zeiss Axio Observer Z1 (Zeiss, Jena, Germany) and quantified in ImageJ.

For migration assays, treated cells were seeded into uncoated Transwell inserts. After 24 h, migrated cells on the lower surface were fixed, stained with crystal violet, imaged, and counted from five random fields per well using ImageJ. For invasion assays, Matrigel was thawed at 4 ℃ overnight, diluted 1:10 in DMEM/F-12, coated onto Transwell inserts, and polymerized at 37 ℃ for 3 h. Cells were then seeded into Matrigel-coated inserts and incubated for 48 h before fixation, staining, imaging, and quantification. For MMP blockade experiments, SCC9 cells were treated with vehicle, IL-12p40, GM6001, or IL-12p40 plus GM6001 during Matrigel-coated Transwell invasion assays.

Colony-formation assay. Cells were seeded in six-well plates at 200 cells/mL (triplicate wells) and cultured for 14 days. Colonies were fixed (4% formaldehyde), stained (0.1% crystal violet), photographed, and counted; only colonies > 1 mm in diameter with > 50 cells were included.

### Animal study

BALB/c nude mice were housed under specific pathogen–free (SPF) conditions at the Animal Experiment Center of Sun Yat-sen University (Guangzhou, China). All procedures were approved by the institutional ethics committee (SYSU-IACUC-2023-000857).

Tumor models. Subcutaneous and orthotopic tongue OSCC models were established according to published protocols with minor modifications [27, 28]. Six-week-old BALB/c nude mice (sex-balanced) were inoculated with SCC7 cells: for subcutaneous tumors, 1 × 10^6^ cells in 100 µL vehicle (DMEM: Matrigel = 1:1) were injected into the right axilla; for orthotopic tumors, 1 × 10^5^ cells in 20 µL vehicle were injected into the right lateral tongue [29].

Treatments. OSCC xenograft tumors were treated with recombinant mouse IL-12p40/IL12B protein (Sino Biological, 51004-M08H; 200 ng per mouse), anti-IL-12p40 antibody (Bio X Cell, BE0051; 50 µg per mouse), or IL-12p40 plus Stattic (APExBIO, 19983-44-9; 400 µg per mouse), administered by intraperitoneal injection every five days. The in vivo Stattic dose was selected within literature-reported effective ranges for STAT3 inhibition in mice, and no overt toxicity (body-weight loss or severe clinical signs) was observed under our conditions [29]. Tumor dimensions were measured every 3 days with calipers, and volumes calculated as V (mm^3^) = ½ × length × width^2^.

Endpoint and tissue processing. Mice were euthanized 28 days after subcutaneous or tongue inoculation. Tumors were excised, weighed, and measured, then fixed, paraffin-embedded, sectioned, and stained with H&E for histopathological evaluation.

### Statistical analysis

Analyses were performed in GraphPad Prism 9.5 and SPSS 26.0. Two groups were compared with unpaired two-tailed Student’s t tests; ≥3 groups with one-way ANOVA plus Tukey’s post hoc test. Tumor growth was analyzed by two-way ANOVA (treatment × time). Categorical data were tested by χ^2^ or Fisher’s exact tests. Survival was assessed by Kaplan-Meier with log-rank tests. ROC/AUC (95% CI) was used for diagnostic performance; Spearman’s ρ for correlations. RNA-seq DEGs were defined as |log_2_FC| ≥ 1 and q < 0.05 (Benjamini-Hochberg). Data are reported as mean ± SEM unless otherwise indicated (biological replicates); *P* < 0.05 was considered significant.

## Results

### Elevated IL-12p40 expression correlates with poor overall survival in OSCC

**Fig. 1:**
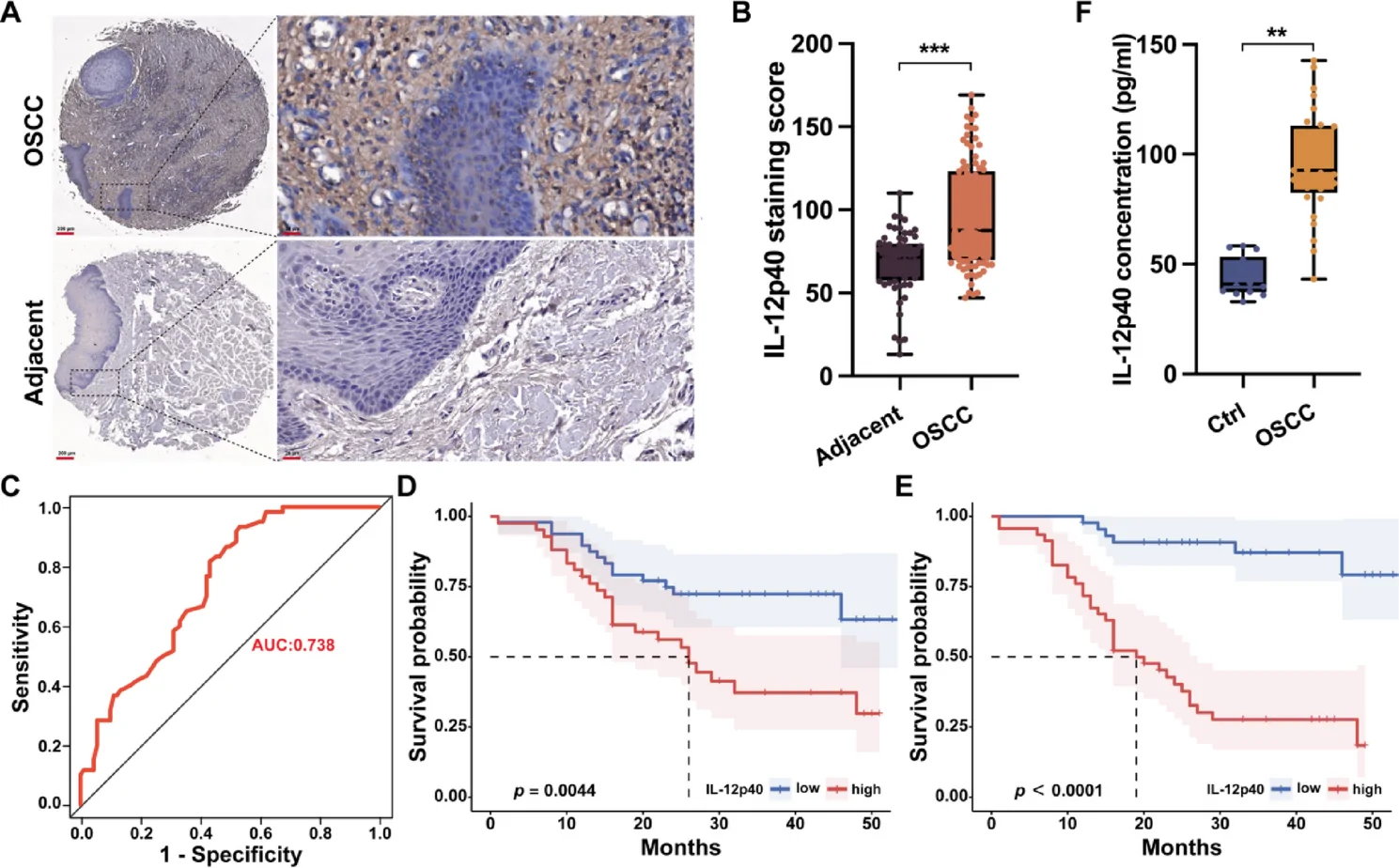
Elevated IL-12p40 expression correlates with poor overall survival in OSCC. (**A**) Representative IHC images of IL-12p40 in OSCC tumor tissues and paired adjacent oral mucosa from tissue microarrays (low and high magnification). (**B**) Quantification of IL-12p40 IHC scores in OSCC versus adjacent normal mucosa (TMA; OSCC n = 90; adjacent n = 60). (**C**) ROC curve evaluating the diagnostic performance of IL-12p40 expression (AUC shown in panel). (**D**) Kaplan-Meier overall-survival curves for malignant-cell IHC high vs. low groups (cutoff as defined in Methods/Table S2). (**E**) Kaplan–Meier overall-survival curves for non-malignant-cell IHC high vs. low groups. (**F**) Serum IL-12p40 concentrations measured by ELISA in OSCC patients versus healthy individuals (OSCC n = 30; healthy n = 15). Scale bars, 200 μm and 20 μm. **P < 0.01; ***P < 0.001

We profiled IL-12p40 by immunohistochemistry (IHC) on OSCC tissue microarrays comprising 90 tumors and 60 matched adjacent oral mucosa from the Third Affiliated Hospital of Sun Yat-sen University (Table S1). IL-12p40 staining was predominantly cytoplasmic in both malignant and non-malignant compartments, with stronger signals overall in tumors. Quantitatively, OSCC tissues showed higher IHC scores (95.71 ± 31.50) than adjacent mucosa (68.27 ± 19.06) (Fig. 1A-B). ROC analysis yielded an AUC of 0.738 (95% CI, 0.660–0.815; *P* < 0.001; Fig. 1C), and a ROC-derived cutoff of 91.0 was used for subsequent stratification (Table S2).

Clinicopathologic correlation analysis showed that IL-12p40 expression in both malignant and non-malignant compartments was associated with lymph-node metastasis (*P* < 0.05; Table S3).

Survival analyses further supported the prognostic value of IL-12p40. Among malignant cells, high IL-12p40 expression was associated with a shorter mean overall survival (32.85 months; 95% CI, 27.354–38.344) and a 3-year survival rate of 43%, whereas the low-expression group showed a longer mean survival (43.30 months; 95% CI, 37.601–48.997) and a 3-year survival rate of 75% (Fig. 1D). A similar pattern was observed for non-malignant cells: the high-expression group had a mean survival of 27.62 months (95% CI, 22.876–32.354) and a 3-year rate of 7%, versus 47.15 months (95% CI, 42.20-52.106) and 84% in the low-expression group (Fig. 1E). Univariate and multivariate Cox regression analyses further showed that high IL-12p40 scores in both malignant and non-malignant compartments were independently associated with poorer 5-year overall survival after adjustment for clinicopathological variables (Table S4).

To extend these observations to the systemic compartment, we analyzed serum from 30 newly diagnosed, treatment-naive OSCC patients and 15 healthy individuals. ELISA showed higher IL-12p40 concentrations in OSCC than in controls (95.59 ± 23.54 pg/mL vs. 43.39 ± 9.42 pg/mL) (Fig. 1F). Taken together, tissue IHC and serum ELISA consistently indicate IL-12p40 elevation in OSCC across compartments.

### IL-12p40 exhibits pro-tumorigenic activity in OSCC

**Fig. 2:**
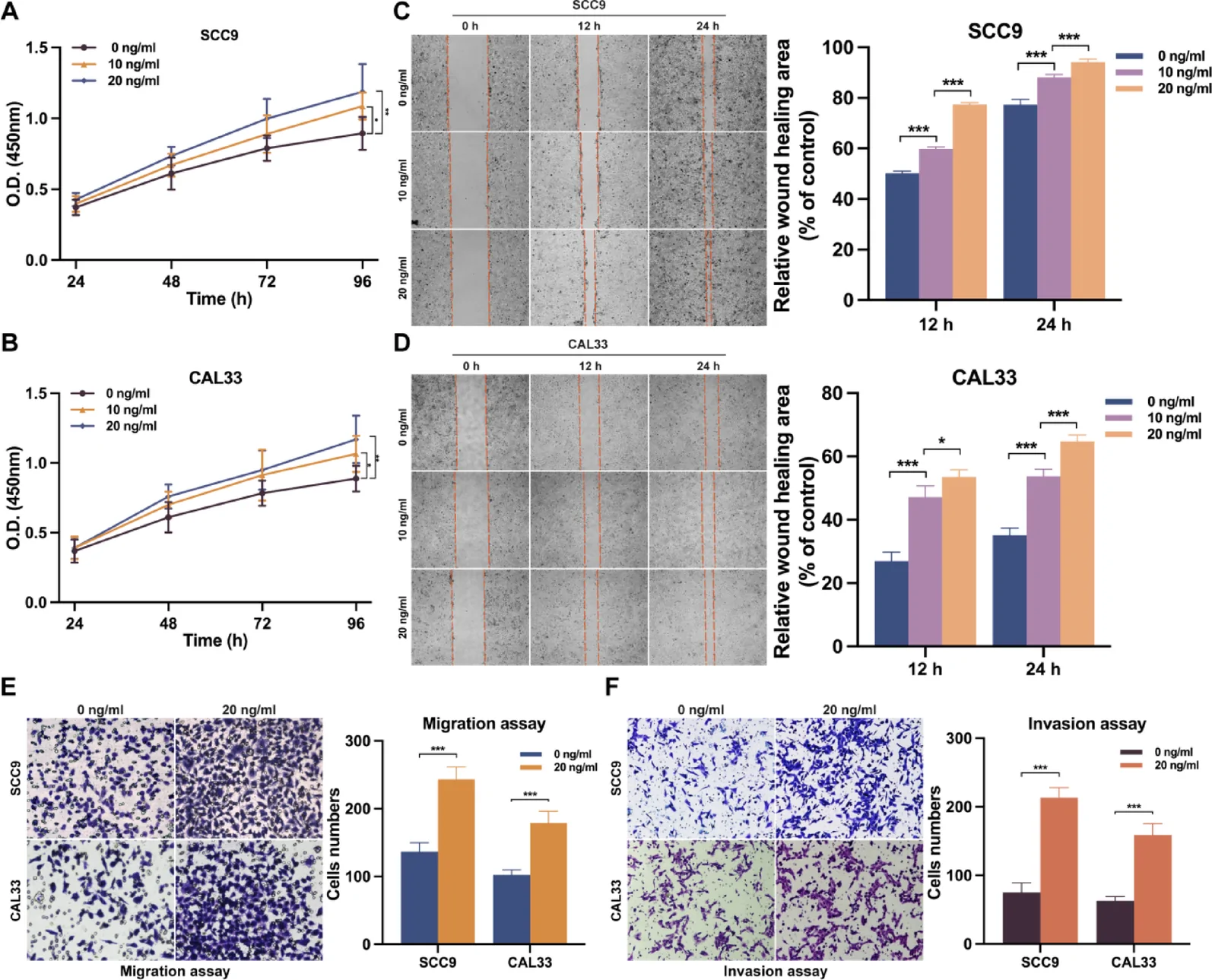
IL-12p40 enhances the proliferative capacity, migratory ability, and invasive potential of OSCC cells. **A**-**B**. Proliferation of SCC9 (**A**) and CAL33 (**B**) treated with the indicated IL-12p40 doses. **C**-**D**. Wound-healing assays in SCC9 (**C**) and CAL33 (**D**) with representative images at 0 h and 24 h and corresponding quantification. E. Transwell migration of OSCC cells after IL-12p40 treatment. **F**. Matrigel-coated Transwell invasion after IL-12p40 treatment. Data are mean ± SEM from ≥ 3 independent experiments. *P < 0.05; **P < 0.01; ***P < 0.001

To define the functional impact of IL-12p40, SCC9 and CAL33 cells were exposed to recombinant IL-12p40 (0–20 ng/mL) and assessed for proliferation, migration, and invasion. IL-12p40 increased cell proliferation in a time- and dose-dependent manner, with the most evident effect observed at 20 ng/mL (Fig. 2A-B). Consistently, IL-12p40 accelerated wound closure and enhanced Transwell migration and Matrigel invasion in both OSCC cell lines (Fig. 2C-F). These data indicate that IL-12p40 promotes OSCC cell growth, motility, and invasive potential *in vitro*.

**Fig. 3:**
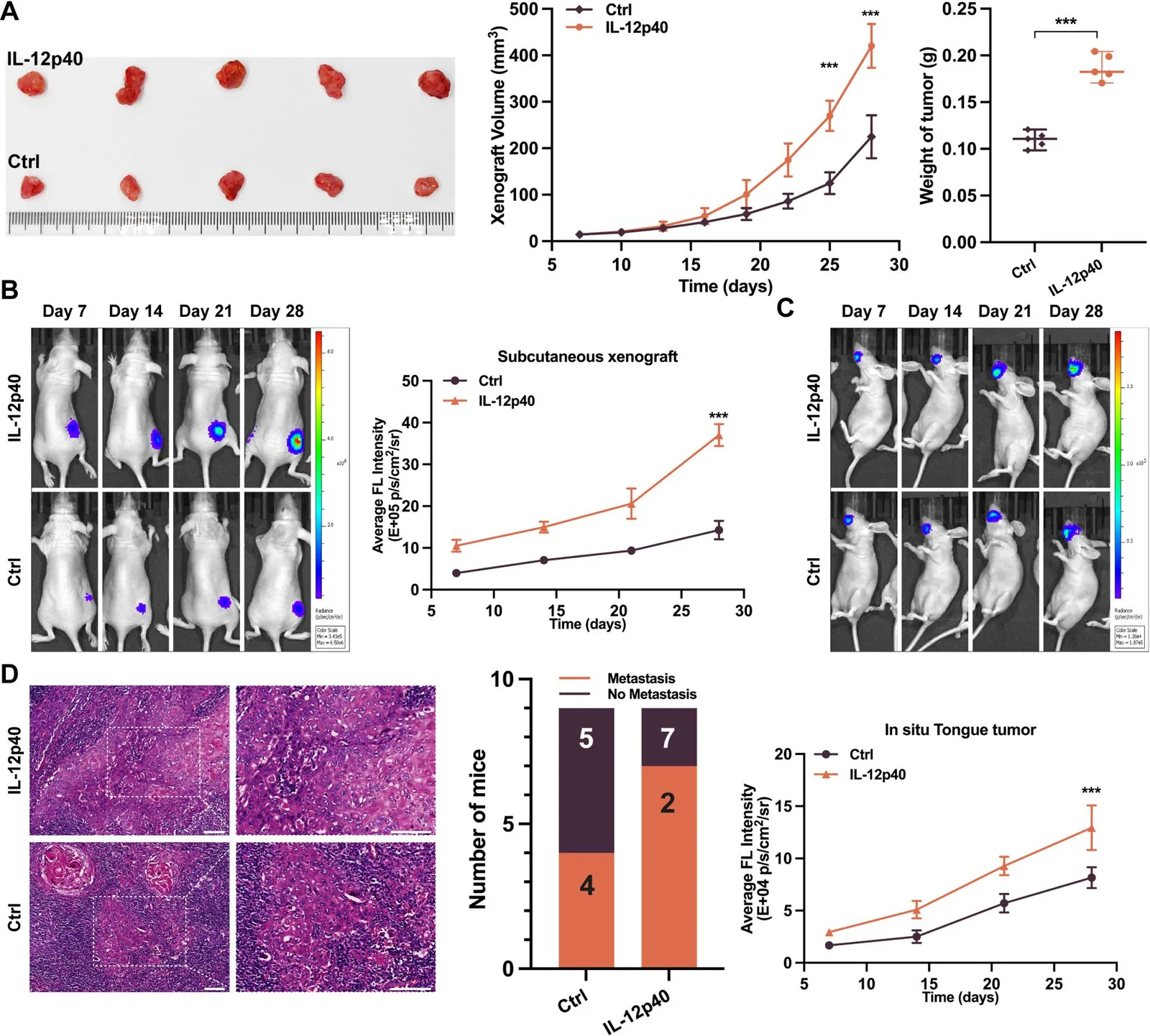
IL-12p40 promotes OSCC tumor growth and lymphatic spread in vivo. **(A)** Representative photographs of xenografts, with growth curves and endpoint tumor weights showing that IL-12p40 enhances SCC7 xenograft growth. **(B)** Representative in vivo fluorescence images of subcutaneous xenografts and quantification of tumor-site signal intensity. **(C)** Representative in vivo fluorescence images of orthotopic tongue tumors and quantification of tumor-site signal intensity. **(D)** Incidence of popliteal lymph-node metastasis in the lymphatic metastasis model. Data are mean ± SEM from ≥ 3 independent experiments. **P < 0.01; ***P < 0.001

In vivo, IL-12p40 treatment increased tumor volume and endpoint tumor weight in subcutaneous xenografts compared with PBS controls (Fig. 3A). In vivo imaging further showed stronger tumor-site fluorescence in both subcutaneous xenografts and orthotopic tongue tumors after IL-12p40 treatment (Fig. 3B-C). Consistently, post-treatment IHC showed stronger IL-12p40 staining in xenograft and orthotopic tumor tissues from IL-12p40-treated mice (Fig. S1A-B). In the popliteal lymph-node metastasis model, IL-12p40 increased nodal involvement, and H&E staining showed larger metastatic deposits occupying a greater proportion of the nodal parenchyma compared with controls (Fig. 3D and Fig. S2). Together, these findings support a pro-tumorigenic role of IL-12p40 in OSCC progression *in vitro* and in vivo.

### IL-12p40 regulates OSCC biology via MMP-linked signaling pathways

**Fig. 4:**
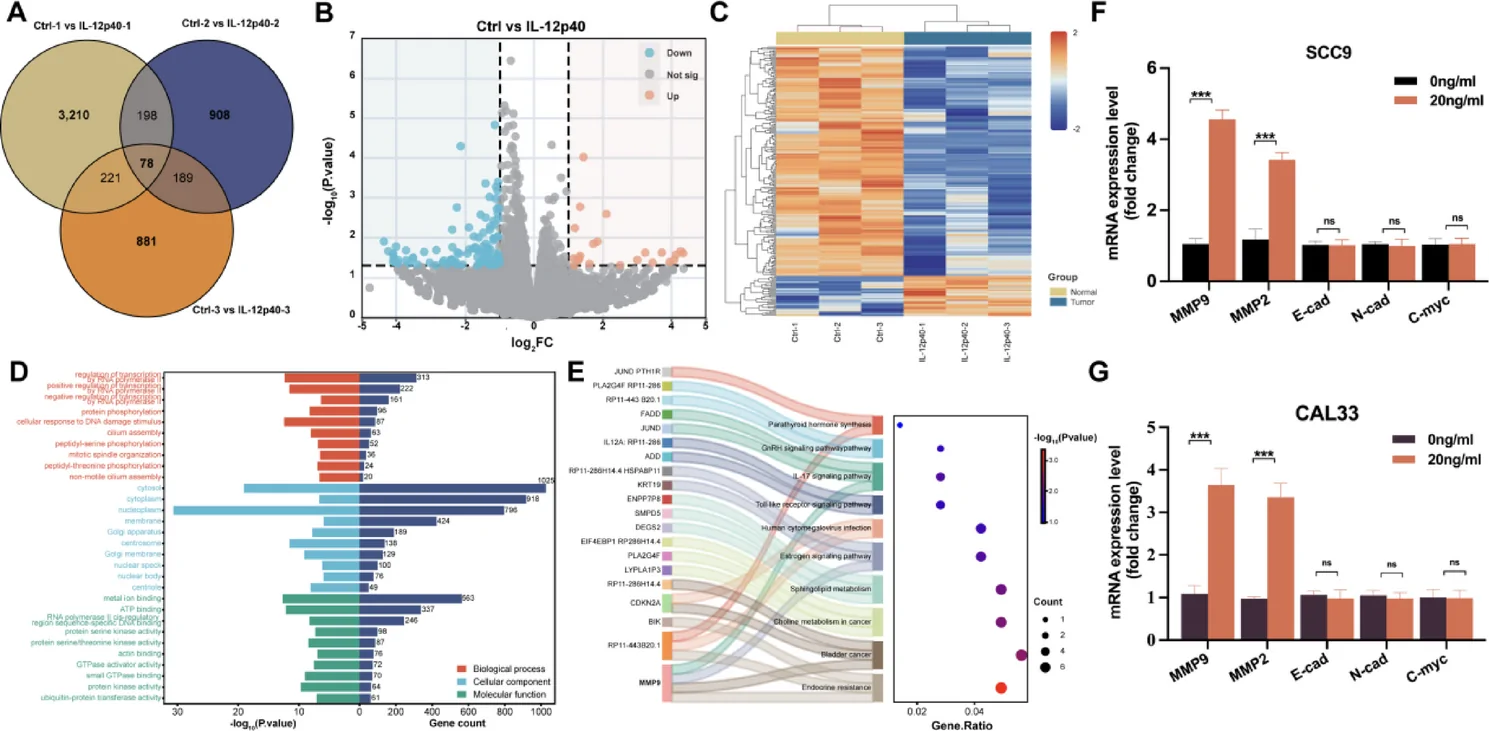
IL-12p40 modulates MMP-linked transcriptional programs in OSCC cells. **(A)** Venn diagram showing overlap of DEGs across three independent SCC9 samples treated with IL-12p40 versus control. **(B)** Volcano plot highlighting significantly upregulated and downregulated DEGs after IL-12p40 stimulation. **(C)** Unsupervised clustering heatmap of DEGs. **(D)** Gene Ontology enrichment of DEGs, grouped by biological process (BP, red), cellular component (CC, blue), and molecular function (MF, green). **(E)** KEGG pathway enrichment of DEGs (Sankey and dot plot; selected pathways shown). **F-G.** qRT-PCR validation of transcripts related to invasion/EMT, including MMP9, MMP2, E-cadherin (E-cad), N-cadherin (N-cad), and C-myc following IL-12p40 stimulation. ns, not significant (*P* ≥ 0.05); ***P < 0.001

To delineate the mechanism of IL-12p40 action in OSCC, we performed RNA-seq on SCC9 cells exposed to recombinant IL-12p40 and conducted three independent control versus treatment comparisons. This analysis identified 265 differentially expressed genes, including 70 upregulated and 195 downregulated transcripts (Fig. 4A-C). Gene Ontology analysis showed enrichment of binding and catalytic activity-related molecular functions, while enriched biological processes included transcriptional regulation, metabolism, and responses to stimuli (Fig. 4D). KEGG enrichment highlighted cancer-related pathways, and MMP9 emerged as a representative gene associated with extracellular-matrix remodeling and cell motility (Fig. 4E; Table S5).

Guided by these transcriptomic signatures, we examined invasion-related transcripts by qRT-PCR. IL-12p40 significantly increased MMP9 and MMP2 expression in both SCC9 and CAL33 cells, whereas E-cadherin, N-cadherin, and C-myc were not significantly altered (Fig. 4F-G). These findings identify MMP2/MMP9 as IL-12p40-responsive downstream readouts and candidate functional mediators in OSCC, providing a basis for subsequent MMP blockade experiments.

### IL-12p40 activates the IL-12Rβ1/STAT3/MMP axis in OSCC cells

**Fig. 5:**
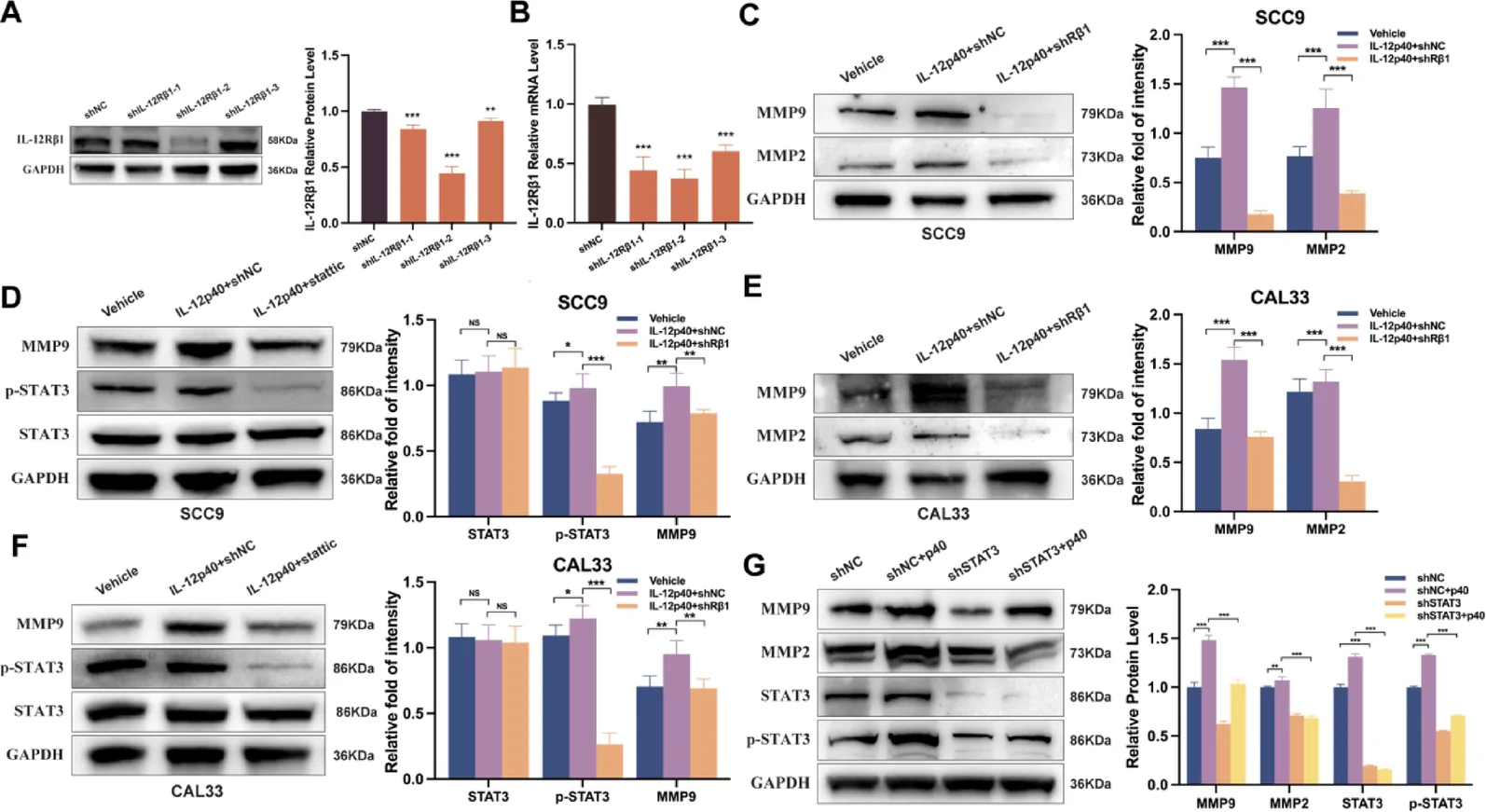
IL-12p40 activates the IL-12Rβ1/STAT3/MMP axis in OSCC cells. **A-B.** IL-12Rβ1 knockdown efficiency in SCC9 (**A**) and CAL33 (**B**) cells, assessed by qRT-PCR and Western blotting. The most effective siRNA was used for subsequent assays. **C-D.** Western blot analysis of MMP9 and MMP2 in IL-12p40-treated SCC9 (**C**) and CAL33 (**D**) cells with or without IL-12Rβ1 silencing. **E-F.** Effects of IL-12p40 and the STAT3 inhibitor Stattic on p-STAT3, MMP9, and MMP2 expression in SCC9 (**E**) and CAL33 (**F**) cells. **G.** Western blot analysis and quantification of STAT3, p-STAT3, MMP2, and MMP9 in shNC- or shSTAT3-transduced SCC9 cells treated with or without IL-12p40. STAT3 knockdown attenuated IL-12p40-induced MMP2/MMP9 upregulation. GAPDH served as the loading control. Data are mean ± SEM from three independent experiments. *P < 0.05; **P < 0.01; ***P < 0.001

IL-12p40 is a receptor-binding subunit that can engage IL-12Rβ1. To determine whether IL-12p40 regulates tumor-cell properties through this receptor, we silenced IL-12Rβ1 in OSCC cells and confirmed efficient knockdown (Fig. 5A-B). We then compared MMP protein levels following IL-12p40 stimulation in control and IL-12Rβ1-knockdown cells. In control SCC9 and CAL33 cells, IL-12p40 increased MMP9 and MMP2 expression, whereas this induction was blunted after IL-12Rβ1 silencing (Fig. 5C-D). These data support the involvement of IL-12Rβ1 in IL-12p40-induced MMP upregulation in OSCC cells.

Because IL-12 family receptors are organized around a JAK-STAT signaling architecture, we next evaluated the involvement of STAT3. Co-treatment experiments showed that IL-12p40 robustly elevated phospho-STAT3 (Y705) and increased MMP levels in both cell lines, whereas the pharmacological STAT3 inhibitor Stattic reduced phospho-STAT3 and concomitantly lowered MMP9 and MMP2 expression (Fig. 5E-F). These pharmacological data, together with the loss of IL-12p40 responsiveness after IL-12Rβ1 knockdown, suggest that STAT3 signaling is involved in the IL-12p40-induced MMP response.

To further strengthen this conclusion using a genetic approach, we established STAT3-knockdown OSCC cells using shRNA. The detailed shRNA screening and lentiviral validation are shown in Fig. S3. In shNC cells, IL-12p40 increased p-STAT3, MMP2, and MMP9 protein levels. In contrast, STAT3 knockdown reduced STAT3 and p-STAT3 levels and attenuated IL-12p40-induced MMP2/MMP9 upregulation (Fig. 5G). Consistently, STAT3 knockdown also weakened IL-12p40-induced wound closure (Fig. S4). These findings provide genetic evidence supporting the functional involvement of STAT3 signaling in the IL-12p40-induced MMP response.

Collectively, receptor perturbation, pharmacological inhibition, and genetic STAT3 knockdown are consistent with a model in which IL-12p40 engages IL-12Rβ1, activates STAT3-associated signaling, and upregulates MMP2/MMP9, thereby contributing to the pro-migratory and pro-invasive phenotypes observed in OSCC cells.

### STAT3 inhibition and MMP blockade attenuate IL-12p40-induced malignant phenotypes in OSCC cells

**Fig. 6:**
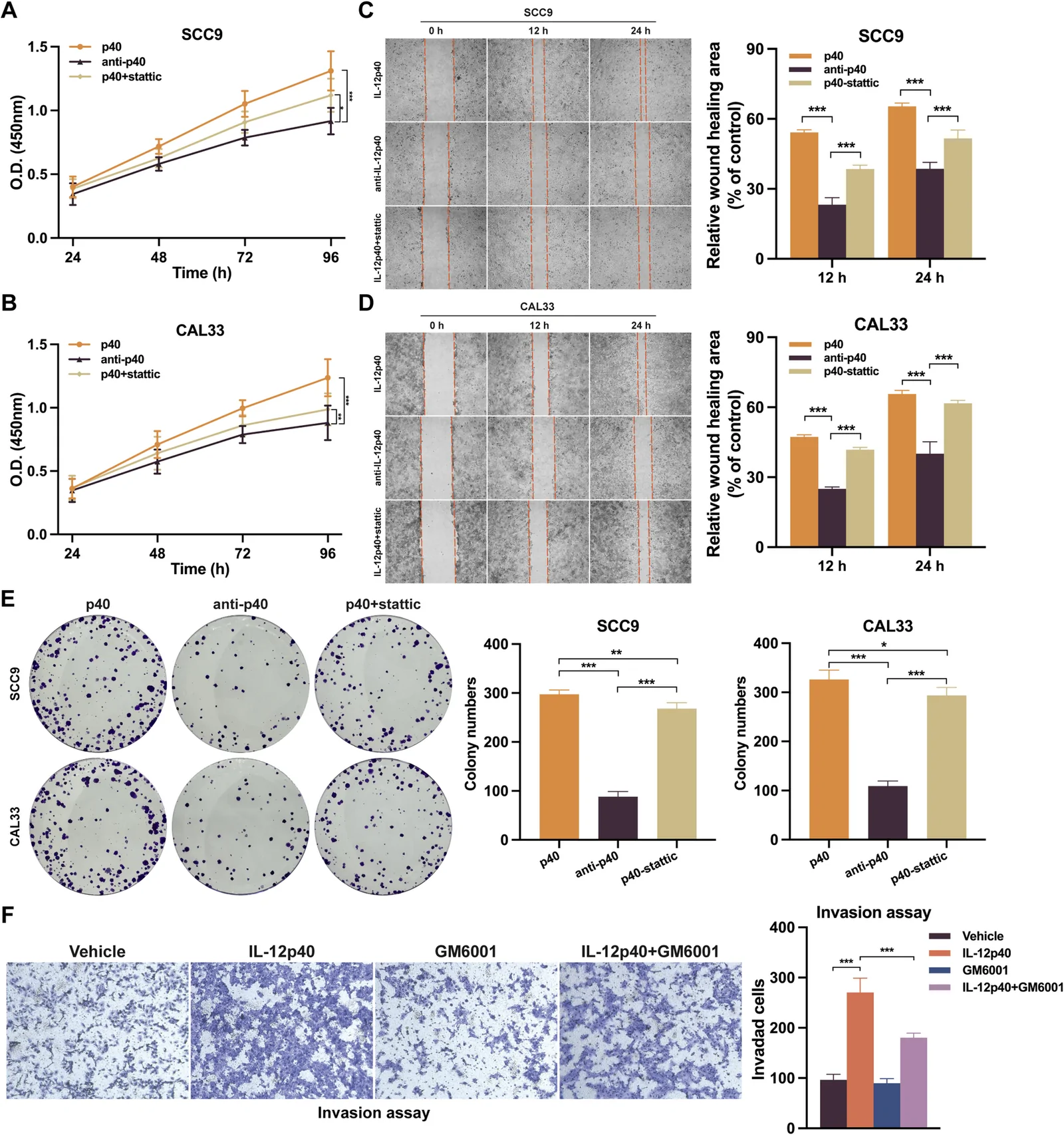
STAT3 inhibition and MMP blockade attenuate IL-12p40-induced malignant phenotypes in OSCC cells. **A-B.** Proliferation of SCC9 (**A**) and CAL33 (**B**) cells treated with IL-12p40, IL-12p40 plus anti-IL-12p40 neutralizing antibody, or IL-12p40 plus the STAT3 inhibitor Stattic. **C-D.** Wound-healing assays in SCC9 (**C**) and CAL33 (**D**) cells under the indicated treatments. Anti-IL-12p40 or Stattic reduced IL-12p40-induced wound closure. **E**. Colony-formation assays in SCC9 and CAL33 cells. Anti-IL-12p40 and Stattic decreased colony formation compared with IL-12p40 alone. **F**. Matrigel-coated Transwell invasion assays in SCC9 cells treated with vehicle, IL-12p40, GM6001, or IL-12p40 plus GM6001. Representative images and corresponding quantification are shown. GM6001 attenuated IL-12p40-induced invasion, supporting the functional contribution of MMP activity to this phenotype. *P < 0.05; **P < 0.01; ***P < 0.001

To probe the functional relevance of the IL-12p40/IL-12Rβ1/STAT3/MMP axis, SCC9 and CAL33 cells were treated with IL-12p40, IL-12p40 plus a neutralizing anti-IL-12p40 antibody, or IL-12p40 plus the STAT3 inhibitor Stattic. CCK-8 assays showed that IL-12p40 increased cell proliferation, whereas anti-IL-12p40 significantly reduced this effect; Stattic likewise attenuated IL-12p40-driven proliferation (Fig. 6A-B). In wound-healing assays, IL-12p40 accelerated wound closure, while either IL-12p40 neutralization or STAT3 inhibition reduced closure relative to IL-12p40 treatment alone (Fig. 6C-D). Consistently, colony-formation assays showed that IL-12p40 enhanced clonogenic growth, which was suppressed by either anti-IL-12p40 or Stattic (Fig. 6E).

Because IL-12p40 induced MMP2/MMP9 expression downstream of STAT3, we next tested whether MMP activity functionally contributes to IL-12p40-induced invasion. Using Matrigel-coated Transwell invasion assays, IL-12p40 markedly increased the number of invaded cells, whereas co-treatment with the broad-spectrum MMP inhibitor GM6001 attenuated this pro-invasive effect (Fig. 6F). These findings support the functional involvement of MMP activity in IL-12p40-induced invasion. Together, these functional assays indicate that IL-12p40 promotes proliferative, migratory, clonogenic, and invasive phenotypes in OSCC cells through a STAT3/MMP-associated signaling program.

### IL-12p40 promotes OSCC tumor growth with STAT3 phosphorylation in vivo.

**Fig. 7:**
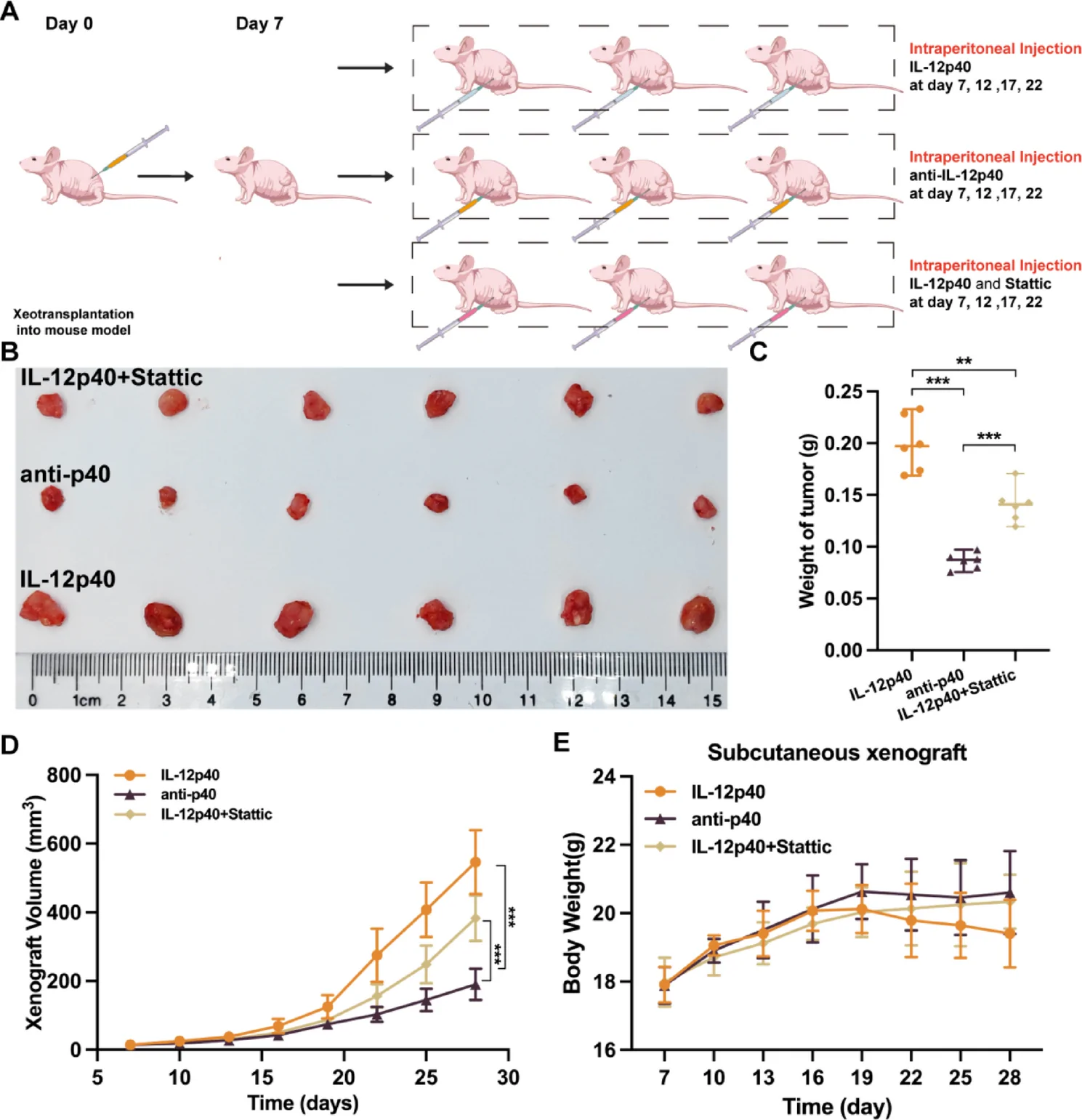
IL-12p40 promotes OSCC tumor growth with STAT3 pathway activation *in vivo*. **A.** Schematic of the OSCC cell-derived xenograft study design and dosing schedule. Mice received intraperitoneal injections of IL-12p40 protein, anti-IL-12p40 neutralizing antibody, or IL-12p40 plus the STAT3 inhibitor Stattic. **B-D.** Representative xenograft photographs (**B**), endpoint tumor weights (**C**), and tumor growth curves (**D**) showing that IL-12p40 increased tumor burden, whereas anti-IL-12p40 or Stattic attenuated this effect. **E.** Body weight monitoring showing no significant differences among treatment groups. Data are mean ± SD (*n* = 6 per group). **P < 0.01; ***P < 0.001

To test the in vivo impact of the IL-12p40/STAT3/MMP axis, we used a subcutaneous xenograft model. Mice received intraperitoneal injections of IL-12p40 protein (200 ng per mouse), anti-IL-12p40 neutralizing antibody (50 µg per mouse), or IL-12p40 plus the STAT3 inhibitor Stattic (400 µg per mouse); tumors were measured every three days (Fig. 7A). Relative to IL-12p40 alone, anti-IL-12p40 significantly reduced xenograft burden, as shown by representative images and endpoint tumor weights (Fig. 7B-C) and growth curves (Fig. 7D). Body weight did not differ among groups (Fig. 7E), and no severe adverse events, including mucositis, weakness, or diarrhea, were observed. Co-administration of Stattic markedly blunted the tumor-promoting effect of IL-12p40, yielding reduced growth kinetics and final tumor mass compared with IL-12p40 alone (Fig. 7B-D).

To corroborate pathway engagement in vivo, we performed IHC for p-STAT3, MMP9, and MMP2 on serial sections from both subcutaneous xenografts and orthotopic tongue tumors. Compared with controls, IL-12p40 treatment increased nuclear p-STAT3 immunoreactivity and enhanced cytoplasmic/pericellular MMP9 and MMP2 expression in both models (Fig. S5A-B). In the neutralization and inhibitor arms, anti-IL-12p40 lowered p-STAT3/MMP signals toward baseline, and IL-12p40 + Stattic similarly reduced these readouts relative to IL-12p40 alone (Fig. S6A-B). These findings mirror our *in vitro* observations and support the involvement of STAT3 phosphorylation and MMP-associated outputs in IL-12p40-related tumor growth in vivo.

## Discussion

In our previous study, IL-12Rβ1 expression was significantly elevated in OSCC tumor tissues, suggesting its potential impact on tumor cell behavior [30]. In the present study, we further showed that IL-12p40 was elevated in OSCC tissues and patient serum, and that higher tissue expression was associated with lymph-node metastasis and poorer survival [31]. Functionally, IL-12p40 promoted OSCC cell proliferation, migration, and invasion *in vitro* and accelerated tumor growth and lymphatic dissemination in vivo. These findings identify IL-12p40/IL-12Rβ1 signaling as a functional contributor to OSCC progression and extend the role of IL-12 family cytokine signaling from immune regulation to tumor-cell-associated malignant behavior.

Mechanistically, transcriptomic profiling and targeted perturbation experiments defined an IL-12Rβ1/STAT3-associated program accompanied by MMP2/MMP9 upregulation in OSCC cells. IL-12Rβ1 silencing reduced IL-12p40-induced MMP expression, while pharmacological STAT3 inhibition attenuated IL-12p40-induced p-STAT3 and downstream MMP signals. Because Stattic is widely used but not fully specific, shRNA-mediated STAT3 knockdown was performed as orthogonal genetic validation and further supported the functional involvement of STAT3 in this pathway. These observations are consistent with the canonical organization of IL-12/IL-23 receptor signaling, in which ligand engagement recruits JAK kinases and activates STAT transcription factors [32, 33]. Within this framework, IL-12p40/IL-12Rβ1 coupling to epithelial STAT programs provides a plausible mechanistic basis for the coordinated induction of p-STAT3 and MMP expression observed in OSCC cells [34, 35].

STAT family members are differentially mobilized by cytokine receptors and show context-dependent activation patterns in solid tumors [36]. IL-12 family ligand binding to IL-12Rβ1 can recruit TYK2 and trigger downstream STAT signaling, and TYK2-dependent steps may influence STAT target selection in oncogenic programs [37, 38]. STAT3 is a key effector in malignant pathways, and TYK2-mediated STAT3 phosphorylation has been implicated in cancer-related signaling [39, 40]. Although STAT1 is commonly linked to antitumor immunity, STAT3 is frequently associated with tumor progression and epithelial-mesenchymal transition [41]. Thus, the IL-12p40/IL-12Rβ1/STAT3 relationship observed here is consistent with broader cytokine-receptor signaling biology and provides a rational framework for understanding how inflammatory cytokine cues may be translated into malignant epithelial programs in OSCC.

MMPs are zinc-dependent endopeptidases that remodel the extracellular matrix and cell-cell junctions, thereby facilitating epithelial tumor invasion and metastasis [42–44]. Under inflammatory conditions, immune, endothelial, and epithelial cells can induce MMP expression through PI3K/MAPK and STAT-regulated transcriptional programs [45, 46]. In this study, IL-12p40 increased MMP2/MMP9 expression, and GM6001-mediated MMP blockade attenuated IL-12p40-induced invasion, supporting the functional involvement of MMP activity in this phenotype. Because many MMP promoters contain STAT3-binding elements, STAT3-dependent transcription provides a mechanistic link between cytokine signaling and metastatic behavior in carcinoma models [47, 48]. Taken together, these findings define a functional IL-12p40–IL-12Rβ1–STAT3/MMP2/MMP9 axis that links inflammatory cytokine signaling to invasive programs in OSCC cells.

**Fig. 8:**
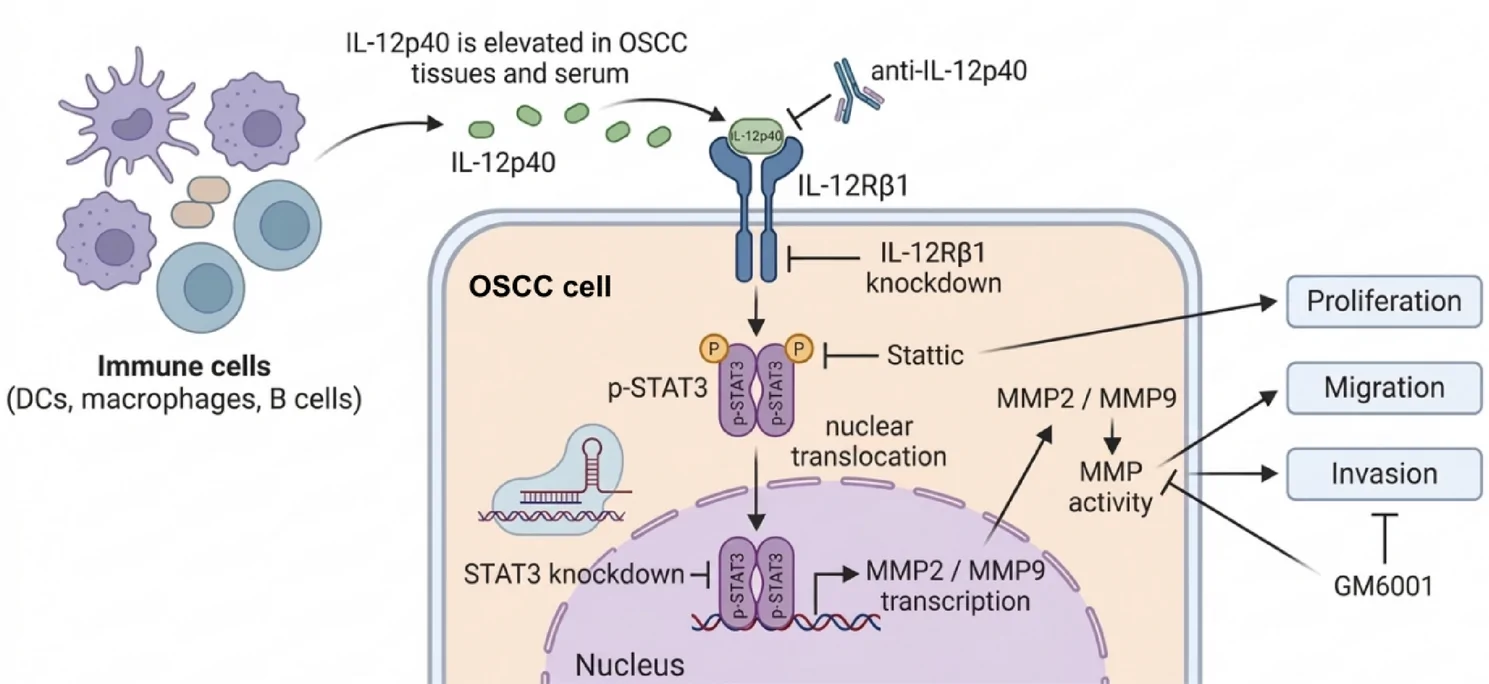
Working model of the IL-12p40–IL-12Rβ1–STAT3/MMP pathway in OSCC. IL-12p40 is elevated in OSCC tissues and serum and is associated with poorer prognosis. In OSCC cells, IL-12p40 engages IL-12Rβ1 and increases STAT3 phosphorylation, thereby upregulating MMP2/MMP9 and supporting proliferation, migration, and invasion. This schematic summarizes the proposed pathway underlying OSCC progression

From a tumor microenvironment perspective, OSCC often arises within a myeloid-rich and immunosuppressive milieu, where cytokine networks can shape epithelial behavior as well as antitumor immunity. Although our experimental design focused primarily on tumor-cell responses to IL-12p40 stimulation, the biological activity of IL-12p40 in OSCC is likely influenced by epithelial–immune–stromal crosstalk. In addition, IL-12p40 may exist as a monomer, homodimer, or as part of IL-12/IL-23 heterodimers, and conventional IHC and ELISA mainly reflect total p40-related signals. These considerations highlight the need for future studies to define the dominant IL-12p40 species in OSCC tissues and to evaluate this pathway in immunocompetent or epithelial–immune co-culture models. Overall, our clinical and experimental observations highlight the IL-12p40–IL-12Rβ1–STAT3/MMP program as a mechanistically supported pathway with potential therapeutic relevance in OSCC (Fig. 8).

### Limitations of the study

This study focused mainly on tumor-cell responses to IL-12p40 stimulation and used immunodeficient mouse models, which may not fully capture immune microenvironment-dependent regulation. Although shRNA-mediated STAT3 knockdown and GM6001-mediated MMP blockade strengthened the mechanistic evidence, rescue experiments and MMP2/MMP9-specific perturbation are still needed to further define the causal hierarchy of this pathway. In addition, IHC and ELISA detect total p40-related signals and cannot distinguish IL-12p40 monomer, homodimer, or IL-12/IL-23 heterodimers. Further validation in immunocompetent models and clinically relevant systems will be required to clarify the translational relevance of this axis.

## Conclusions

In summary, IL-12p40 is elevated in OSCC tissues and serum and is associated with lymph-node metastasis and poorer survival. Functionally, IL-12p40 promotes OSCC malignant phenotypes *in vitro* and tumor progression in vivo. Mechanistically, IL-12p40 engages IL-12Rβ1 and activates STAT3-associated signaling with MMP2/MMP9 upregulation. STAT3 knockdown and GM6001-mediated MMP blockade further support the functional involvement of the STAT3/MMP program. These findings support an IL-12p40–IL-12Rβ1–STAT3/MMP-related pathway in OSCC progression and provide a rationale for further therapeutic validation.

## Supplementary Information

Below is the link to the electronic supplementary material.

Supplementary Material 1.

## Data Availability

The data that support the findings of this study are available from the corresponding author upon reasonable request.

## Abbreviations

OSCC: Oral squamous cell carcinoma
TIME: Tumor immune microenvironment
IL-12p40: Interleukin-12p40
IL-12Rβ1: Interleukin-12 receptor beta 1
STAT3: Signal transducer and activator of transcription 3
p-STAT3: Phosphorylated STAT3
MMP: Matrix metalloproteinase
IHC: Immunohistochemistry
ELISA: Enzyme-linked immunosorbent assay
qRT-PCR: Quantitative real-time polymerase chain reaction
RNA-seq: RNA sequencing
DEG: Differentially expressed gene
GO: Gene Ontology
KEGG: Kyoto Encyclopedia of Genes and Genomes
ROC: Receiver operating characteristic
AUC: Area under the curve
HR: Hazard ratio
CI: Confidence interval
CCK-8: Cell Counting Kit-8
