# Supplementary material for: IL-12p40 drives oral squamous cell carcinoma progression via MMPs and IL-12Rβ1/STAT3 signaling
Source: Cancer Cell Int. 2026 Jul 16;26:335. doi: 10.1186/s12935-026-04413-0 (PMC13656609; doi:10.1186/s12935-026-04413-0)
Supplement: Supplementary file 1 [file 12935_2026_4413_MOESM1_ESM.docx]

**Supplementary Material**

**Figure S1**

**Figure S1. IL-12p40 immunohistochemistry in mouse tumor tissues.**

**A-B**. Representative IHC for IL-12p40 in subcutaneous xenografts (A) and orthotopic tongue tumors (B) from PBS control and IL-12p40-treated mice. Compared with PBS, the IL-12p40 group shows stronger cytoplasmic staining in malignant epithelial cells and peri-tumoral stroma. Images were taken from serial sections; Scale bar, 100 μm.

**Figure S2**

**Figure S2. H&E staining of metastatic popliteal lymph nodes in the mouse model.**

Representative H&E sections of popliteal lymph nodes from control (PBS) and IL-12p40-treated mice. Compared with control, the IL-12p40 group shows larger metastatic deposits occupying a greater proportion of the nodal parenchyma; Scale bar, 100 μm.

**Figure S3**

**Figure S3.** The knockdown efficiency of STAT3 was identified at protein and RNA levels using western blotting (A) and qRT-PCR (B). **P* < 0.05; ***P* < 0.01; ****P* < 0.001.

**Figure S4**

**Figure S4. STAT3 knockdown attenuates IL-12p40-induced wound closure in OSCC cells.**

**A.** Representative wound-healing images of shNC- or shSTAT3-transduced OSCC cells treated with or without IL-12p40. **B.** Quantification of wound closure. STAT3 knockdown weakened the pro-migratory effect induced by IL-12p40. Data are shown as mean ± SEM from three independent experiments. ****P* < 0.001.

**Figure S5**

**Figure S5. IHC staining for p-STAT3, MMP9 and MMP2 in mouse tumor tissues in animal experiments.**

**A.** Representative IHC staining for p-STAT3, MMP9 and MMP2 in subcutaneous xenograft tumor tissues. **B.** Representative IHC staining for p-STAT3, MMP9 and MMP2 in orthotopic tongue tumor tissues; Scale bar, 100 μm.

**Figure S6**

**Figure S6. IHC staining for p-STAT3, MMP9, and MMP2 in mouse tumor tissues from animal experiments.**

**A-B.** Representative IHC images of p-STAT3, MMP9, and MMP2 in two subcutaneous xenograft tumors from each group, selected to represent the consistent expression patterns observed across all mice within the group (n = 6 per group). Scale bars, 100 μm.

**Table S1. Clinicopathological features of OSCC and normal oral mucosa specimens.**

| **Characteristic** | **Level** | **OSCC（n=90）** | **Normal（n=60）** |
| --- | --- | --- | --- |
| Age (years) |  |  |  |
|  | Mean (Range) | 58.7 (23-88) | 56.6 (27-86) |
| Gender |  |  |  |
|  | Male | 64 | 44 |
|  | Female | 26 | 16 |
| Smoke |  |  |  |
|  | Yes | 55 | 21 |
|  | No | 35 | 39 |
| Drink |  |  |  |
|  | Yes | 27 | 9 |
|  | No | 63 | 51 |
| Betelnut |  |  |  |
|  | Yes | 11 | 4 |
|  | No | 79 | 56 |
| Clinical Stage |  |  |  |
|  | C_I_ | 33 | — |
|  | C_II_ | 29 | — |
|  | C_III_ | 20 | — |
|  | C_IV_ | 8 | — |
| T stage |  |  |  |
|  | T1 | 36 | — |
|  | T2 | 37 | — |
|  | T3 | 12 | — |
|  | T4 | 5 | — |
| N stage |  |  |  |
|  | N0 | 65 | — |
|  | N1 | 10 | — |
|  | N2 | 13 | — |
|  | N3 | 2 | — |
| Lymph |  |  |  |
|  | Positive | 25 | — |
|  | Negative | 65 | — |

**Table S2. ROC curve analysis of IL-12p40 in the clinical samples.**

|  | **Cut-off value** | **Sensitivity** | **Specificity** | **Youden Index** | **AUC** | **95% CI** | ***P*** |
| --- | --- | --- | --- | --- | --- | --- | --- |
| **OSCC vs Normal** | 91.0 | 46.7% | 93.3% | 0.40 | 0.738 | 0.660–0.815 | <0.001 |

**Table S3. Statistical analysis of IHC staining scores of IL-12p40 in clinical sample.**

| **Characteristics** | | **malignant cells** | | ***P*** | **non-malignant cells** | | ***P*** |
| --- | --- | --- | --- | --- | --- | --- | --- |
|  |  | Low | High |  | Low | High |  |
| **Gender** | Male | 28 | 36 | 0.645 | 31 | 33 | 0.389 |
|  | Female | 10 | 16 |  | 10 | 16 |  |
| **Age**  **(years)** | ≤60 | 19 | 22 | 0.469 | 21 | 20 | 0.324 |
|  | ＞60 | 19 | 30 |  | 20 | 29 |  |
| **Tumour**  **stage** | T_1+2_ | 30 | 43 | 0.654 | 33 | 40 | 0.890 |
|  | T_3+4_ | 8 | 9 |  | 8 | 9 |  |
| **LN**  **metastasis** | Negative | 32 | 33 | **0.030*** | 34 | 31 | **0.038*** |
|  | Positive | 6 | 19 |  | 7 | 18 |  |
| **Clinical**  **stage** | C_I+II_ | 30 | 32 | 0.078 | 28 | 34 | 0.911 |
|  | C_III+IV_ | 8 | 20 |  | 13 | 15 |  |
| **Pathological**  **grade** | Well | 4 | 9 | 0.612 | 5 | 8 | 0.158 |
|  | Moderate | 19 | 26 |  | 25 | 20 |  |
|  | Poor | 15 | 17 |  | 11 | 21 |  |
| **Clinical stage was determined according to the 7th edition of the UICC-AJCC TNM staging system**. *P* values were calculated using Pearson’s chi-square test. **P <* 0.05. | | | | | | | |

**Table S4. Univariate and multivariate Cox regression analyses of factors associated with 5-year overall survival in patients with OSCC**

| **Variable** | **Univariate analysis** | |  | **Multivariate analysis** | |
| --- | --- | --- | --- | --- | --- |
|  | **HR (95% CI)** | ***P*** |  | **HR (95% CI)** | ***P*** |
| **Age**  **(≤60 vs＞60)** | 1.61  (0.84-3.07) | 0.150 |  | 1.67  (0.76-3.69) | 0.206 |
| **Gender**  **(male vs female)** | 1.12  (0.56-2.20) | 0.754 |  | 0.58  (0.27-1.23) | 0.156 |
| **T classification**  **(T_1+2_ vs. T_3+4_)** | 1.16  (0.53-2.53) | 0.714 |  | 1.34  (0.62-3.01) | 0.473 |
| **LN metastasis**  **(LN− vs. LN+)** | 2.13  (1.11-4.10) | **0.023*** |  | 2.34  (1.20-4.56) | **0.013*** |
| **Pathological grade**  **(well vs. moderate;**  **well vs. poor)** | 0.71  (0.26-1.95)  0.74  (0.37-1.46) | 0.503  0.381 |  | 0.74  (0.27-2.05)  0.70  (0.34-1.34) | 0.563  0.255 |
| **Malignant Cell Score**  **(high vs. low)** | 2.44  (1.19–5.00) | **0.015*** |  | 2.35  (1.14–4.84) | **0.020*** |
| **Non-malignant Cell Score**  **(high vs. low)** | 4.74  (2.16–10.44) | **<0.001***** |  | 4.56  (2.07–10.05) | **<0.001***** |
| **HR, hazard ratio; CI, confidence interval; LN, lymph node.** **P <*  0.05; ****P* < 0.001. | | | | | |
|  | | | | | |

| **Signaling pathways** | ***P*** | **Representative DEGs** |
| --- | --- | --- |
| Endocrine resistance | 0.000 | ***MMP9****; RP11-443B20.1; BIK* |
| Bladder cancer | 0.003 | ***MMP9****; CDKN2A; RP11-286H14.4* |
| Choline metabolism in cancer | 0.004 | *LYPLA1P3; PLA2G4F; EIF4EBP1 RP286H14.4* |
| Sphingolipid metabolism | 0.006 | *DEGS2; SMPD5; ENPP7P8* |
| Estrogen signaling pathway | 0.012 | ***MMP9****; KRT19; RP11-286H14.4 HSPA8P11* |
| Human cytomegalovirus infection | 0.018 | *RP11-443B20.1; CDKN2A* |
| Toll-like receptor signaling pathway | 0.023 | *ADD; IL12A: RP11-286* |
| IL-17 signaling pathway | 0.012 | ***MMP9****; JUND; FADD* |
| GnRH signaling pathway | 0.024 | *RP11-443 B20.1; PLA2G4F RP11-286* |
| Parathyroid hormone synthesis, secretion and action | 0.081 | *RP11-443B20.1; JUND PTH1R* |

**Table S5. Top ten enriched signaling pathways and representative DEGs in IL-12p40-treated SCC9 cells**

**Table S6. siRNA and shRNA sequences used in this study**

| **siRNA** | **sequence** |
| --- | --- |
| IL-12Rβ1-1 | GGAGGTCACTTACCGACTA |
| ***IL-12Rβ1-2** | GTCACACTCTGGGTGGAAT |
| IL-12Rβ1-3 | GCTCTAGCGGCTTGTCTAA |

*** indicates the sequence selected for subsequent assays.**

| **Name** | **Target gene** | **Sequence** |
| --- | --- | --- |
| shNC | Non-targeting control | TTCTCCGAACGTGTCACGT |
| shSTAT3-1 | STAT3 | CGGCAACAGATTGCCTGCATT |
| ***shSTAT3-2** | STAT3 | CAGCCTCTCTGCAGAATTCAA |
| shSTAT3-3 | STAT3 | CAGCTGAACAACATGTCATTT |

*** indicates the sequence selected for subsequent assays.**

**Table S7. The primers for qRT-PCR**

| **Name** | **Sequence** |
| --- | --- |
| **IL-12p40** | sense: 5'-ATGGCCGTCTCAGCACTTC-3'  antisense: 5'-AGGCAACTCTCGGTTGACATC-3' |
| **IL-12R**β**1** | sense: 5'-AGCTGCGTATGGAGTGGGA-3'  antisense: 5'-GAGGCAGGACTCAGTATCACA-3' |
| **MMP9** | sense: 5'-TGTACCGCTATGGTTACACTCG-3'  antisense: 5'-GGCAGGGACAGTTGCTTCT-3' |
| **MMP2** | sense: 5'-CCCACTGCGGTTTTCTCGAAT-3'  antisense: 5'-CAAAGGGGTATCCATCGCCAT-3' |
| **E-cad** | sense: 5'-CGAGAGCTACACGTTCACGG-3'  antisense: 5'-GGGTGTCGAGGGAAAAATAGG-3' |
| **N-cad** | sense: 5'-AGCCAACCTTAACTGAGGAGT-3'  antisense: 5'-GGCAAGTTGATTGGAGGGATG-3' |
| **C-myc** | sense: 5'-GGCTCCTGGCAAAAGGTCA-3'  antisense: 5'-CTGCGTAGTTGTGCTGATGT-3' |
| **RPLP0** | sense: 5'-GGCGACCTGGAAGTCCAACT-3'  antisense: 5'-CCATCAGCACCACAGCCTTC-3' |
| **GAPDH** | sense: 5'-TGAGAATGAAGGCTGAGGTGT-3'  antisense: 5'-GCAGCGACCATCCAGTCATT-3' |

**Table S8. Key antibodies, recombinant proteins, and inhibitors used in this study**

| **Reagent** | **Source** | **Catalog No.** | **Application** | **Dilution / concentration** | **Notes** |
| --- | --- | --- | --- | --- | --- |
| Recombinant human IL-12p40 / IL12B protein | Sino Biological | 10052-H08H | *In vitro* stimulation | 20 ng/mL | Purity >95%; endotoxin <1.0 EU/μg; used for cell assays |
| Recombinant mouse IL-12p40 / IL12B protein | Sino Biological | 51004-M08H | *In vivo* stimulation | 200 ng/mouse | Purity >95%; endotoxin <1.0 EU/μg; used in mouse tumor models |
| Anti-IL-12p40 antibody for IHC/WB | Abcam | ab106270 | IHC/WB | IHC 1:150;  WB 1:1000 | Primary antibody for IL-12p40 detection |
| Anti-human  IL-12/IL-23 p40 neutralizing antibody | R&D Systems | AF309 | *In vitro* neutralization | 1 μg/mL | Used for *in vitro*  IL-12p40 neutralization assays |
| Anti-mouse  IL-12p40 neutralizing antibody | Bio X Cell | BE0051 | *In vivo* neutralization | 50 μg/mouse | Neutralizing antibody used in mouse tumor treatment |
| Stattic | APExBIO | A2224 / CAS 19983-44-9 | STAT3 inhibition | 10 μM *in vitro*;  400 μg/mouse i*n vivo* | Small-molecule STAT3 inhibitor |
| GM6001 | APExBIO | A4050 | MMP inhibition | 10 μM | Broad-spectrum MMP inhibitor |
| IL-12Rβ1  antibody | Affinity | DF2508 | WB | 1:1000 | Primary antibody |
| STAT3  antibody | Bioworld | AP0366 | WB | 1:800 | Primary antibody |
| p-STAT3  antibody | Bioworld | BS4180 | WB/IHC | WB 1:1000;  IHC 1:100 | Primary antibody |
| MMP9  antibody | Affinity | AF5228 | WB/IHC | WB 1:1000;  IHC 1:100 | Primary antibody |
| MMP2  antibody | Affinity | AF5330 | WB/IHC | WB 1:800;  IHC 1:100 | Primary antibody |
| GAPDH  antibody | CST | #2118 | WB | 1:1000 | Loading control |
